# A serendipitous product of the reaction of famotidine with copper(II)

**DOI:** 10.1107/S2056989026003804

**Published:** 2026-04-24

**Authors:** Aylen Grenni, Bruno Rosa, Javier Ellena, Gianella Facchin, Natalia Alvarez

**Affiliations:** a Química Inorgánica, Departamento Estrella Campos, Facultad de Química, Universidad de la República, Montevideo, Uruguay; b Instituto de Física de São Carlos, Universidade de São Paulo, IFSC - USP, 13566-590, São Carlos, SP, Brazil; National Taras Shevchenko University of Kyiv, Ukraine

**Keywords:** crystal structure, copper(II) complex, Hirshfeld surface analysis

## Abstract

Reaction of CuCl_2_ and the anti-ulcer drug famotidine in aqueous solution results in hydrolytic cleavage of the organic mol­ecules with loss of sulfonamide fragments and subsequent crystallization of a new Cu complex, di­chlorido­{3-[(2-guanidino­thia­zol-4-yl)methyl­sulfan­yl]propanoic acid}copper(II) monohydrate, [CuCl_2_(C_8_H_12_N_4_O_2_S_2_)]·H_2_O (**1**). The Cu^II^ ions adopt a square-pyramidal environment with the basal plane comprising one chloride ion and the organic ligand acting as the N,N,S-donor bis-chelate tridentate group. The second chloride ion resides in the apical position and it completes the electrically neutral complex. The supra­molecular landscape is dominated by conventional hydrogen bonding involving strong hydrogen-bond donors and acceptors [O(N)H⋯O and O(N)H⋯Cl], which assemble the complex units and solvent water mol­ecules into bilayers parallel to the *bc* plane. The primary significance of the contacts with H atoms was assessed with the results of Hirshfeld analysis, which suggest a 51.3% contribution of such contacts to the surface of individual complex mol­ecules. The relatively short chalcogenide bonds [S⋯O = 3.006 (2) Å] and NH_2_ (guanidine)-π inter­actions are also relevant to the packing. The chemical identity of **1** and the bulk sample is confirmed by elemental analysis, IR spectroscopic and thermogravimetric data.

## Abstract 2

The reaction of copper(II) chloride with famotidine in aqueous solution at 333 K gave the title compound, which features coordination of the thio­propanoic acid derivative obtained in the acid-catalyzed *in situ* hydrolysis of famotidine.

## 1. Chemical context

During the COVID pandemic many researchers found themselves looking to contribute to the search of active compounds against the coronavirus. One of the strategies used was based on drug repositioning. In this context, H_2_ receptor antagonists were targeted as potential active drugs. Famotidine is an example of the latter, since it is generally used to reduce gastric acid secretion by blocking histamine action on parietal cells and is also given before surgery to lower the risk of postoperative nausea and aspiration pneumonia (Brunton *et al.*, 2018 ▸). Moreover, famotidine is a IV class drug (low solubility-low permeability) and this has attracted attention for studies on its solid form modification, as well as for strategies exploiting the coordination potential of the famotidine mol­ecule, which bears a set of donor atoms for bonding with metal ions. In particular, during the 1990s potentiometric studies confirmed the ligand properties of famotidine (Kozłowski *et al.*, 1992 ▸; Kubiak *et al*., 1996 ▸). In a subsequent search of new metal–famotidine complexes, an improved pharmacological effect was found with coordination to Zn^II^ (Arya *et al*., 2010 ▸). A family of famotidine complexes is useful for other treatments. For instance, Zn^II^ (Amin *et al*., 2010 ▸), Cu^II^ (Kozłowski *et al.*, 1992 ▸) and Co^III^ complexes (Miodragović *et al.,* 2006 ▸) were deemed to be potential anti­fungal and anti­microbial agents.
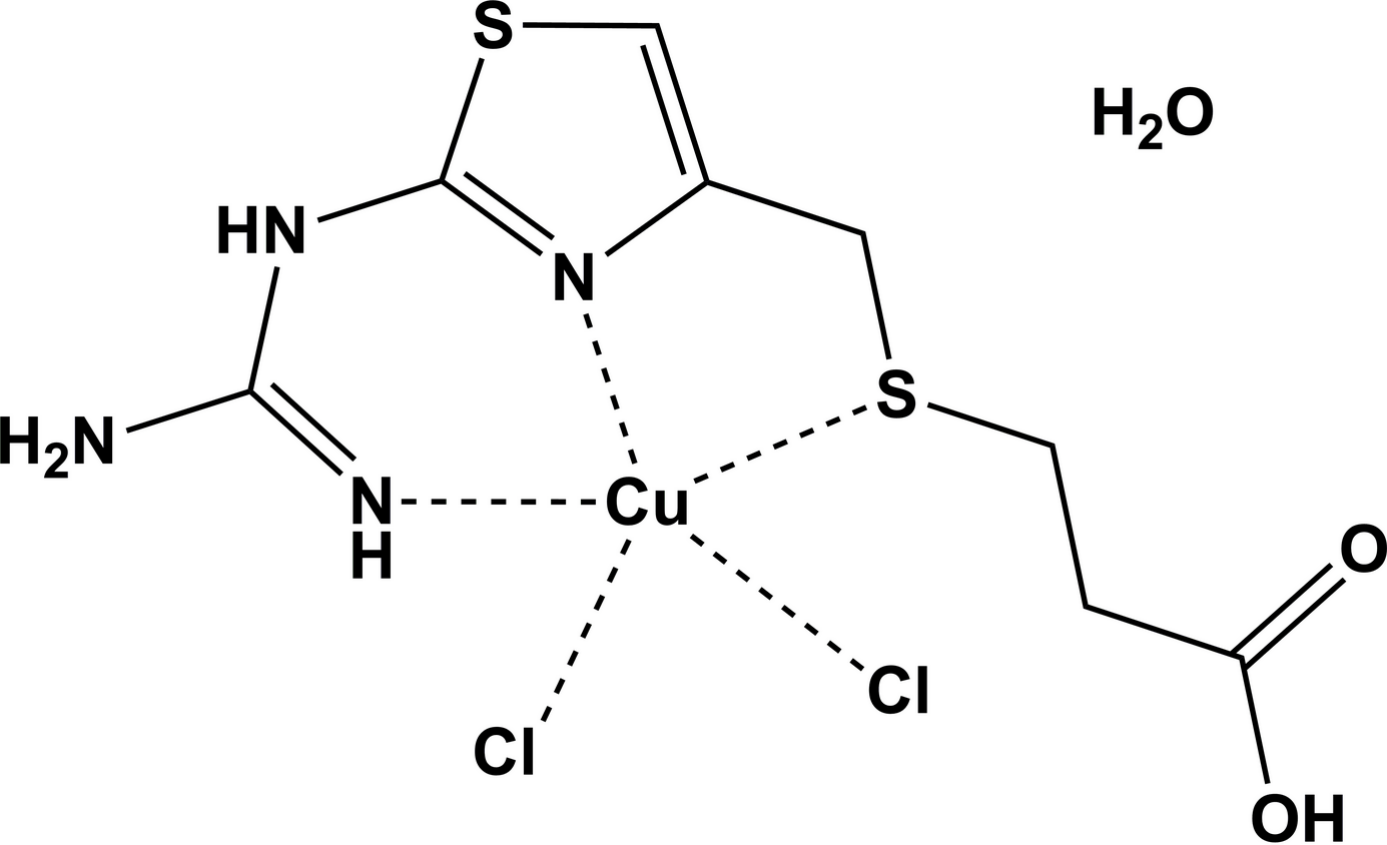


Following these studies, we have designed a synthetic procedure to obtain a new coordination compound containing famotidine and copper(II). However, even under relatively mild conditions, the reaction at 333 K for 45 min and at a pH of 3, gave a copper(II) complex with the acid-catalyzed hydrolysis product of famotidine, namely the corresponding (alkyl­thio)­propanoic acid obtained by a nucleophilic attack at the sulfamoyl group. This acid is expected to be one of the degradation products under gastric conditions (Suleiman *et al*., 1989 ▸). The hydrolysis products of famotidine have been deemed inactive as H_2_ antagonists (Saikia *et al.*, 2019 ▸) and have never been structurally characterized. However, the accumulation of these degradation products on waste and natural waters have become a problem, making it necessary to find alternatives to treat contaminated waters (Karpińska *et al*., 2010 ▸; Molla *et al*., 2017 ▸). Structural knowledge of these degradation products contributes to the development of more efficient materials for the removal of these persistent contaminants.

We describe here the crystal structure of the title compound **1** with an emphasis on performing a critical comparative analysis with the available Cu^II^ (Kubiak *et al.*, 1996 ▸) and Ni^II^ (Russo *et al.*, 2021 ▸) complexes with famotidine ligands. Although the hydrolysis degradation product of famotidine in stomach conditions has already been described (Suleiman *et al.*, 1989 ▸), there are no structural reports on such particular species or the coordination compounds thereof.

## 2. Structural commentary

**Figure 1:**
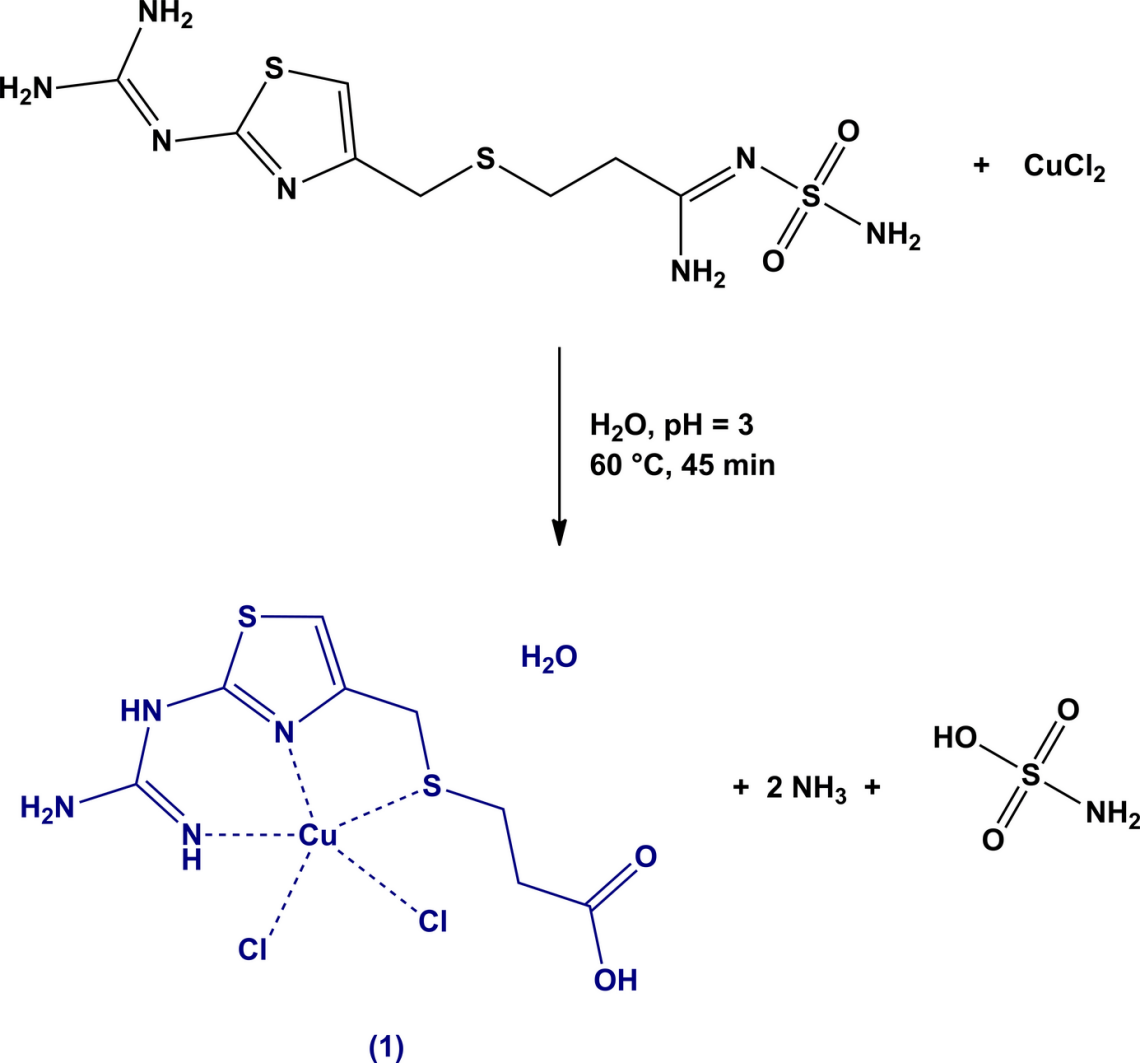
Synthetic scheme for the obtained Cu^II^ complex involving *in situ* hydrolytic fragmentation of famotidine.

**Figure 2:**
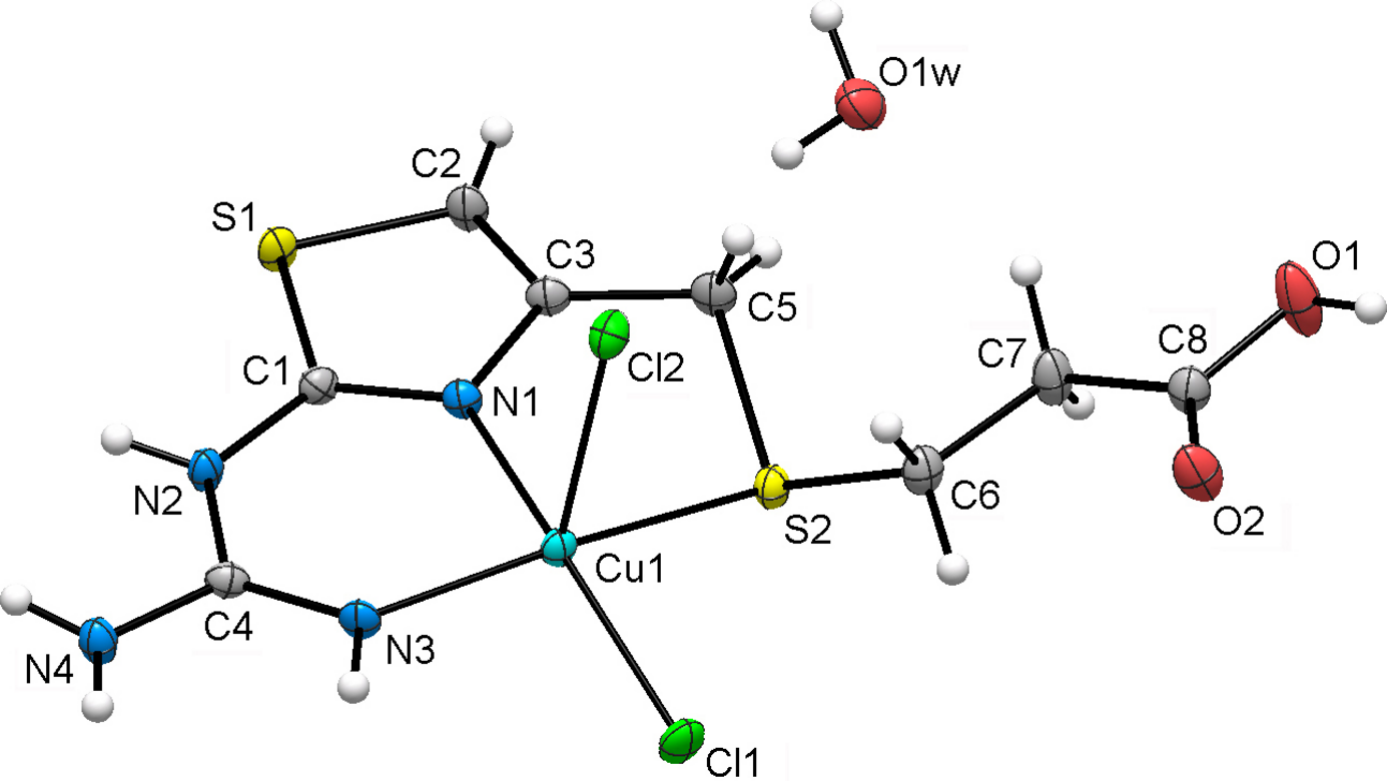
Mol­ecular structure of **1** showing displacement ellipsoids drawn at 50% probability level and the atom-labeling scheme.

Previous reports on the behavior of copper(II) in aqueous solution with famotidine at low pH (Kozłowski *et al.*, 1992 ▸; Kubiak *et al.*, 1996 ▸) indicated the formation of a stable 1:1 complex with an electrically neutral famotidine coordinated in a tetra­dentate manner through aminic-N, thia­zolic-N, sulfide-S and amidinic-N atoms (Kubiak *et al.*, 1996 ▸). Under similar conditions we were able to obtain a copper(II) complex containing a neutral famotidine hydrolysis product (Fig. 1 ▸), coordinated in a tridentate manner through the terminal aminic-N, thia­zolic-N and sulfide-S atoms. The asymmetric unit with numbering scheme for non-H atoms is presented in Fig. 2 ▸. The copper(II) atom in **1** adopts a square-pyramidal coordination geometry, as indicated by the low τ value of 0.12, which is close to the value of zero expected for an idealized square pyramid (Addison *et al.*, 1984 ▸). The four basal positions are occupied by the ligand and atom Cl1 whereas the second chloride Cl2 resides at the apex of the pyramid. This may be compared with the structure of the Cu^II^ perchlorate complex with famotidine, where the metal ion coordination exhibits a square-planar geometry fully completed with the organic ligand N- and S-donors (Kubiak *et al.*, 1996 ▸).

Further insights into the electronic and protolytic features of the ligand are possible when comparing selected bond distances with those for the related famotidine (polymorph A; CSD refcode: FOGVIG08; Saikia *et al.*, 2019 ▸) and its Cu^II^ complex (TAWVIW; Kubiak *et al.*, 1996 ▸) (Table 1 ▸). First, it is worth mentioning that the C—N distances in the guanidine moiety change due to a charge and proton redistribution (Russo *et al.*, 2021 ▸). In the famotidine structure, both N3 and N4 are doubly protonated and singly bonded to C4, whereas N3 is deprotonated due to the formation of a double bond with C4. This attribution is slightly nominal due to the evident contribution of charge-separated canonical forms, *e.g*. N2^−^—C4=N4H_2_^+^, as evidenced by a perceptible Bader charge at the N2 atom in famotidine [−1.04; Overgaard & Hibbs, 2004 ▸]. However, the electronic effects imposed by the coordination are clearly visible. Upon coordination, N4 stays doubly protonated; meanwhile, N2 and N3 are both monoprotonated. The electronic effect is reflected in the shortening of the N3—C4 distance and the perceptible increase in the N2—C4 distance. The N1—C1 distances are similar in the organic mol­ecule and both Cu^II^ complexes, and as well the coordination through the sulfide-S atom has only minor reflectance in the bond lenghts of the sulfur–aliphatic linkage. For the outer C—NH_2_ group of the coordinated guanidine fragment, the corresponding N4—C4 bond is also less sensitive to the coordination, being actually the same for **1** and the parent famotidine [1.346 (4) and 1.3425 (4) Å, respectively]. One can postulate that this fragment retains certain double bonding C4 N4 and a partial positive charge, which are inherent to the guanidine structure.

## 3. Spectroscopic and thermogravimmetric analysis

**Figure 3:**
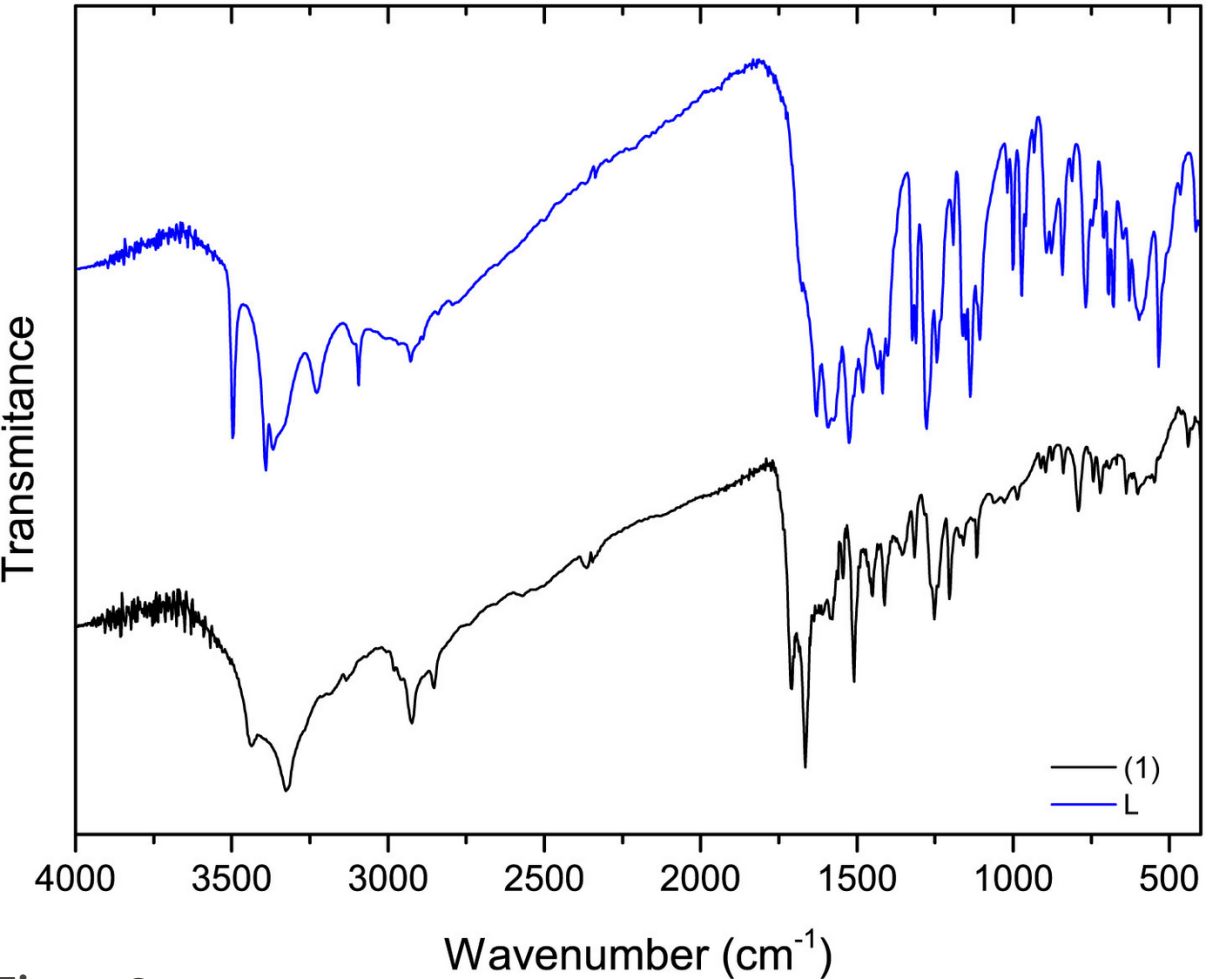
FTIR spectra for famotidine (black) and **1** (blue).

In order to ascertain the chemical identity of **1** and the bulk sample, we have performed elemental analysis, infrared spectroscopy and thermogravimetric studies. The IR spectra of **1** (Fig. 3 ▸) were analysed by comparing with the previous literature data for a detailed FTIR study of famotidine based on DFT results (Sagdinc *et al.*, 2005 ▸) and reports on Ni^II^–famotidine complexes (Russo *et al.*, 2021 ▸).

There is sufficient evidence for the ligand structure, its difference from the parent famotidine and its coordination fashion in **1**. The most relevant features in the IR spectra of **1** include:

· The appearance of carbonyl stretching bands together with the disappearance of bands related to the SO_2_ group in the sulfonyl fragment in famotidine. Strong sharp peaks at 1710 cm^−1^ and 1252 cm^−1^ for **1** correspond to the C=O and C—O stretching modes and their appearance confirms the formation of a terminal carboxyl­ate group. Bands reflecting stretching of the S—O bonds at 1150, 720 and 640 cm^−1^ for famotidine, disappear in the spectra of **1**.

· Modifications in the bands related to the NH_2_ groups in guanidine and sulfonyl fragments. The bands at 3230 cm^−1^ related to NH_2_ and CH_2_ stretching that can be assigned to the sulfonyl moiety, disappear in the spectra of **1**. At the same time, the bands at 3500 and 3390 cm^−1^ in famotidine, which make a large contribution to the vibrational modes of the amino groups both in the guanidine and sulfonyl moieties, yield an absorption centered at 3440 cm^−1^ for **1**. This is consistent with the changes discussed for the C4—N3 and N2 bond distances upon coordination. The observed decrease in intensity may be attributed to the loss of the sulfonyl moiety in **1**.

· Thia­zole and sulfide band modifications. The stretching of the CH_2_—S2 bond is reflected by bands at 2925 cm^−1^ in famotidine and 2850 cm^−1^ in **1**, evidencing the coordination through S2. The C—N stretching in the guanidine fragment goes from 1680 cm^−1^ in famotidine to 1710 and 1660 cm^−1^ in the complex. Deformation vibrational modes in the thia­zole fragment move from 1280 and 820 cm^−1^ in famotidine to 1250 and 790 cm^−1^, respectively, in **1**. The small shift in frequencies is consistent with the observed variations in bond distances in the thia­zole group upon coordination.

**Figure 4:**
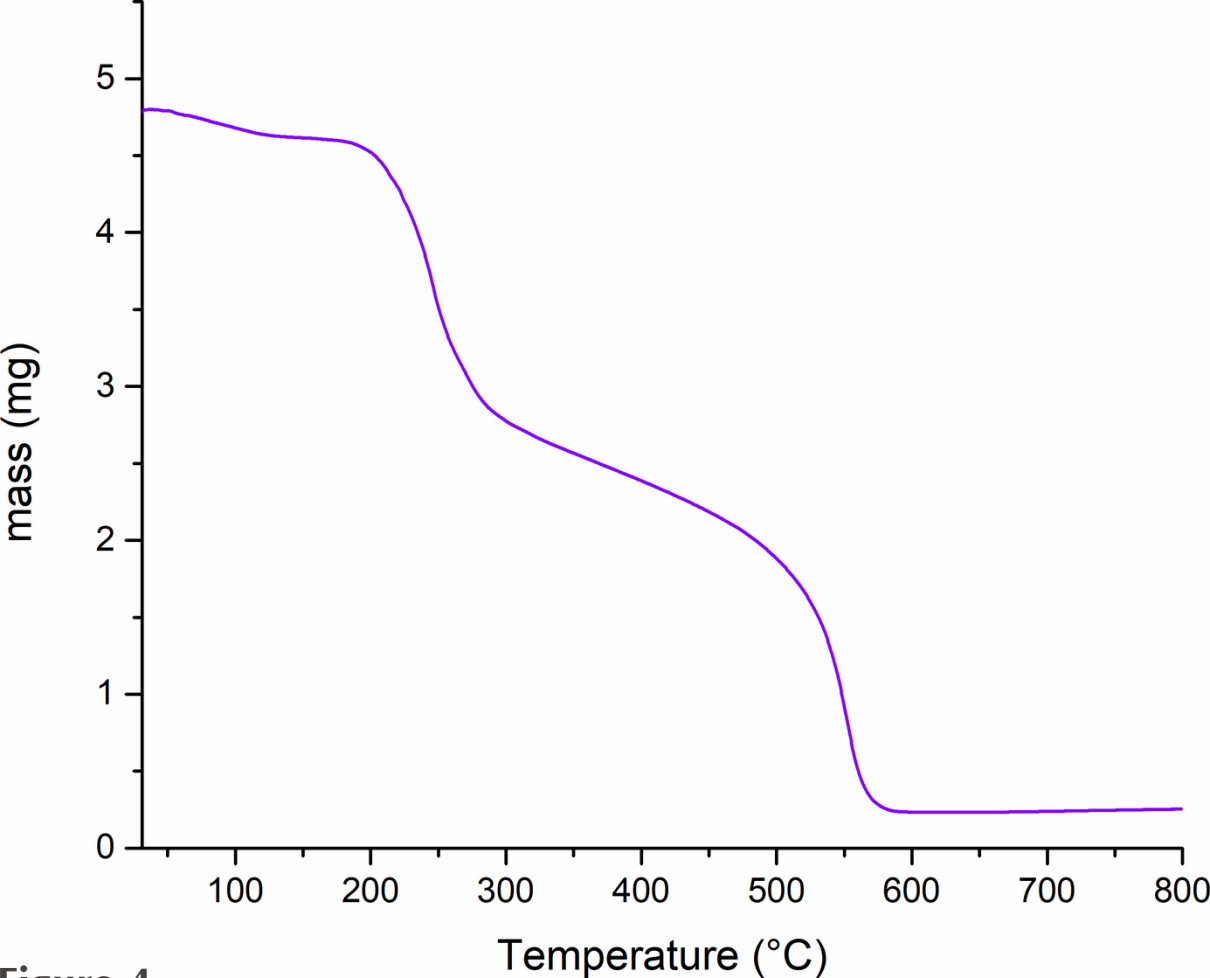
TGA curve corresponding to **1**.

Thermal stability and water content for the obtained solid were assessed through thermogravimetry. Fig. 4 ▸ presents the TGA plot for **1**, with the weight loss including three distinct steps. The first step, from 303 to 457 K, corresponds to 4.18% mass loss and it indicates elimination of the solvate water mol­ecules (calculated 4.36%). The second step, in the range of from 457–673 K, coincides with the weight loss of 42.64%. It may be ascribed to the rupture of the S2—C3 bond and consequent loss of a thiopropionate fragment of the ligand (calculated 45.85%), similarly to the previously reported Ni^II^–famotidine complex (Russo *et al.*, 2021 ▸). Further decomposition of the remaining metal-organic material is observed above 770 K.

## 4. Database survey

A Cambridge Structural Database (CSD, Version 2023.3.1; Groom *et al.*, 2016 ▸) search for coordination compounds containing the guanidine-thia­zole fragment yielded four results, which are copper(II) famotidine complex (TAWVIW; Kubiak *et al.*, 1996 ▸), a monodeprotonated famotidine cobalt(III) complex (XELLEG; Miodragović *et al.*, 2006 ▸) and two nickel(II) complexes (BEVQAV01, ODAFEI; Russo *et al.*, 2021 ▸). In all cases, the famotidine was coordinated through one terminal aminic-N atom, a thia­zole-N atom and a sulfide-S atom, similarly to the main famotidine fragment preserved by the organic ligand in the structure of **1**. A detailed comparative analysis of the effect of the differences in the ligand with the copper(II)–famotidine crystal structure (TAWVIW) in the supra­molecular inter­actions landscape is presented in the next section. There are no entries with the products of hydrolytic degradation of famotidine or their metal complexes in the CSD.

## 5. Supra­molecular features and comparative Hirshfeld surface analysis

With a rich set of conventional hydrogen-bond donors and acceptors, the structure is maintained by an intricate and extended hydrogen-bonding landscape involving the carboxyl­ate groups, the solvate water mol­ecules, the coordinated chlorides and aminic-N atoms that is listed in Table 2 ▸.

**Figure 5:**
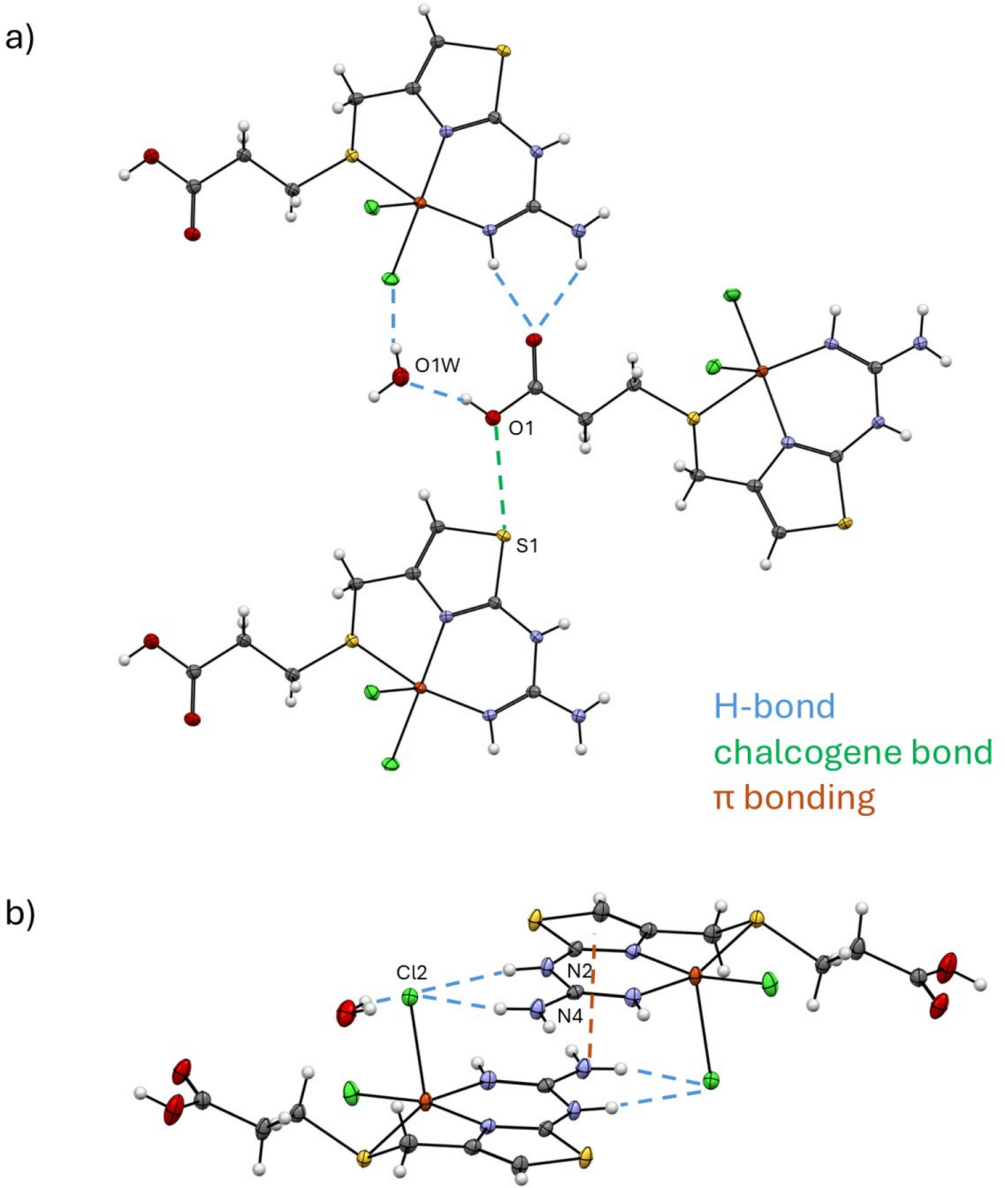
Principal inter­molecular inter­actions in the structure of **1**: (*a*) fragment of the layer parallel to the *bc* plane, with a set of H-bond and chalcogen inter­actions and (*b*) Reciprocal hydrogen-bonding and NH_2_/thia­zole stacking inter­actions, which result in an anti-aligned packing with the formation of the bilayers.

It is possible to distinguish the hydrogen-bonded layers parallel to the *bc* plane that pack in an anti-aligned fashion forming a bilayer. Fig. 5 ▸(*a*) presents a view along the *a* axis with inter­molecular inter­actions indicated as dashed lines. No π–π inter­actions involving solely the thia­zole moieties were found in the structure, similarly to the pattern present in the copper(II)–famotidine complex (Kubiak *et al.*, 1996 ▸). The inter­molecular inter­actions driving the formation of the single layer are centered mostly in the carb­oxy­lic acid group of the ligand. The carbonyl-O atom acts as a bifurcated acceptor for the N3—H2*N*⋯O2^ii^ and N4—H4*N*⋯O2^ii^ bonds involving the guanidine aminic-NH donors of a contiguous complex [symmetry code: (ii) *x*, −*y* + 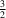, *z* + 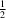]. The corresponding geometry parameters are consistent with a medium strength hydrogen bond [N⋯O distances are 3.002 (3) and 3.020 (3) Å; Desiraju & Steiner, 1999 ▸]. A much stronger bond is associated with the acidic H atom and aqua acceptor, with a corresponding O1⋯O1*W*^i^ distance of 2.577 (3) Å and nearly straight angle at the H atom [symmetry code: (i) −*x* + 1, −*y* + 1, −*z* + 1].

A remarkable feature of the structure is the presence of a chalcogen-type S⋯O inter­action involving the thia­zole-S atoms. Such chalcogenide sites are usually electron deficient and produce directional σ-holes that can give rise to a special kind of inter­molecular inter­action with the most negatively polarized atoms. The systematic study of chalcogen inter­actions has produced a set of expected geometrical descriptors. They are distances below the sum of the van der Waals radii (3.35 Å for S and O atoms) and an O⋯S—C_aromatic_ angle of about 160 ° (Zhang *et al.*, 2015 ▸), which perfectly fit the observed parameters for **1**: S1⋯O1 = 3.007 (2) Å and C1—S1⋯O1 = 164.87 (11) °. No such contacts are present in famotidine itself. This may further indicate the impact of thia­zole N-coordination on the distribution of the π-electron density, which enhances the ability of S atoms for hypervalent bonding.

The function of the apical chloride ion Cl2 as a multiple hydrogen-bond acceptor is shown in Fig. 5 ▸(*b*). It is not surprising that the number of conventional hydrogen-bond inter­actions in this case is larger than the one found for the equatorial chloride Cl1 (three and two, respectively), since the weakly coordinated Cl2 is more underbonded and basic. All these NH⋯Cl2 and OH⋯Cl2 inter­actions are relatively strong and directional (Table 2 ▸), being the primary forces for sustaining pairwise association of two inversion-related complexes (symmetry code: −*x* + 1, −*y* + 1, −*z* + 2) and subsequent maintenance of the bilayer structure. This association is also favorable for the formation of reciprocal stacking, with the guanidine N4 atoms situated exactly above the neighboring thia­zole ring centroids *Cg* at 3.246 (3) Å and with the angle subtended by the N4⋯*Cg* axis to the ring normal being 2.8 (2)°. We regard this remarkable inter­action as a kind of cation–π bonding (Yamada, 2020 ▸), which involves positively polarized C4 N4H_2_^δ+^ fragment of guanidine and thia­zole as the efficient π-donor. For comparison, in the famotidine polymorph B, the π-cloud of thia­zole functions as an acceptor of C—H⋯π hydrogen bonds (Overgaard & Hibbs, 2004 ▸).

**Figure 6:**
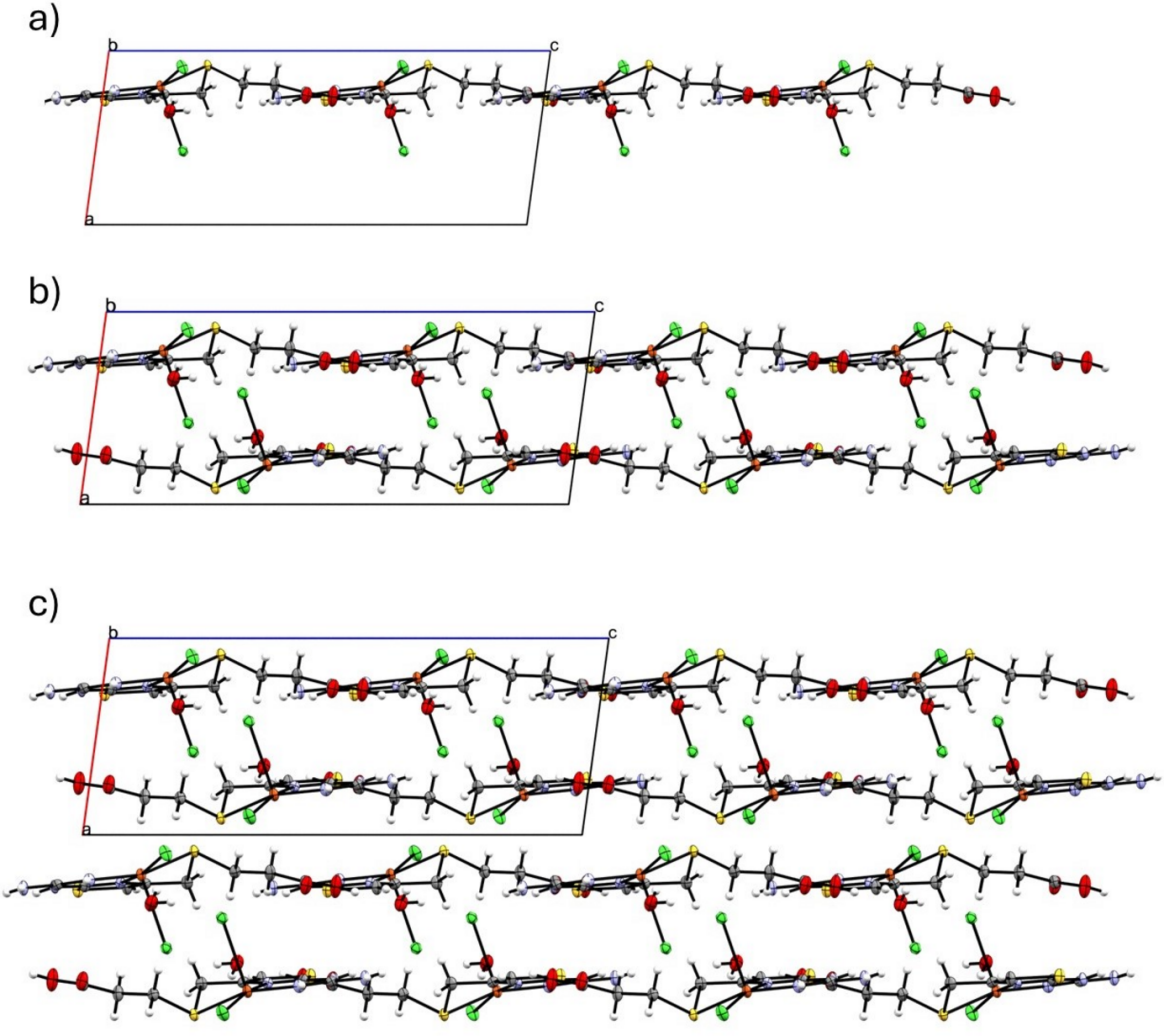
Structure of **1** viewed down the *b* axis showing a single layer in the *bc* plane (*a*), the formation of the bilayer by dense stack of two anti-aligned layers (*b*) and the packing of the bilayers (*c*).

The adjacent bilayers are related by translation along the *a*-axis direction in the crystal and are separated by *ca* 6.89 Å [*i.e.* asin(180 – β)]. There are no strong conventional inter­actions between the bilayers, while the shortest observed contacts represent typical weak hydrogen bonding C5—H5*B*⋯Cl1(−*x* + 2, *y* − 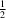, −*z* + 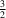) = 3.560 (3) Å, with the angle at the H-atom of 142 (3)° (Desiraju & Steiner, 1999 ▸), which engages the most polarized and acidic (thia­zole-4)CH_2_ hydrogen atoms (Fig. 6 ▸).

**Figure 7:**
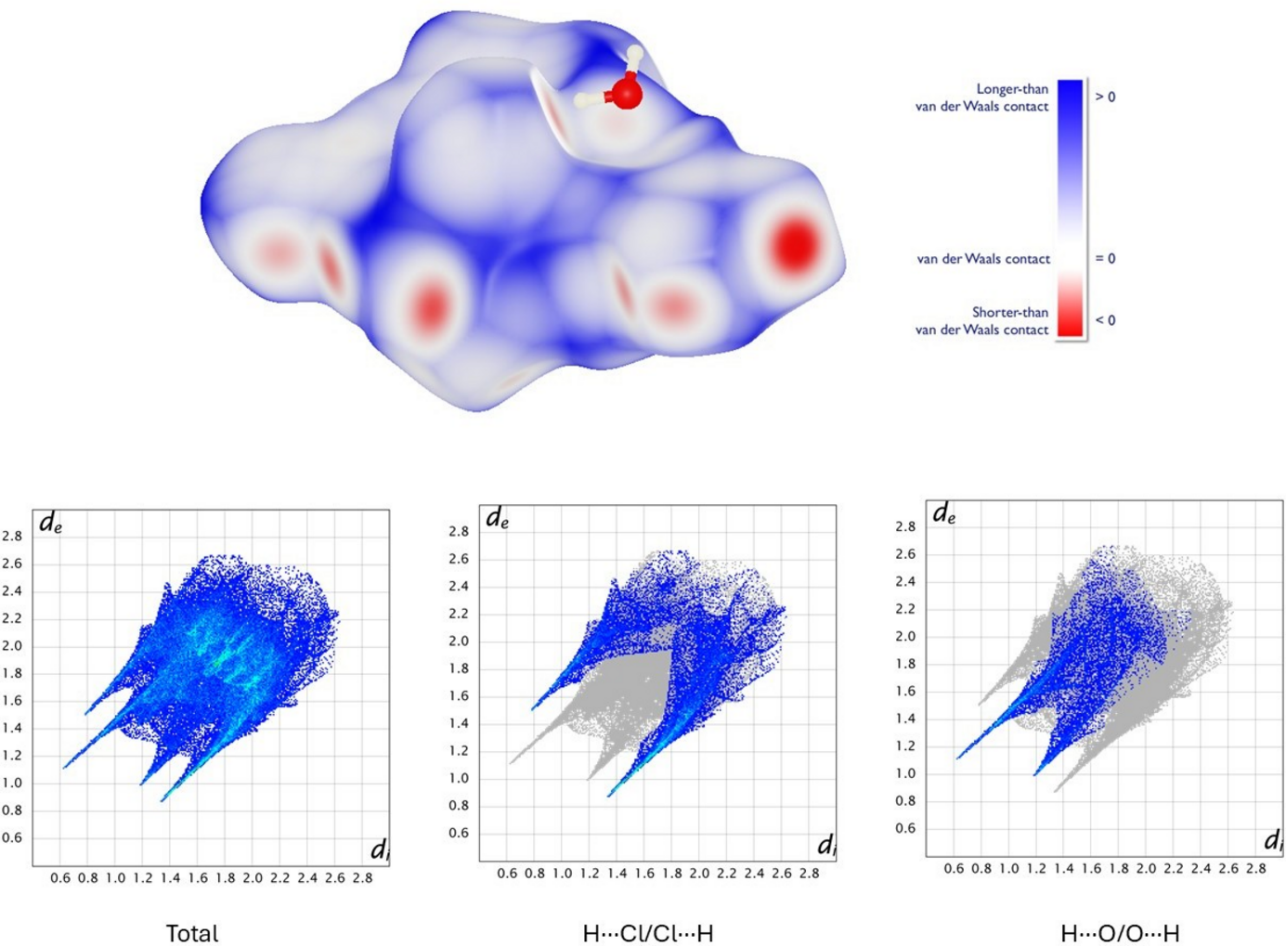
Hirshfeld surface mapped with *d*_norm_ around the copper(II) complex in **1** (top) and two-dimensional fingerprint plots for all inter­actions in the structure, and H⋯Cl/Cl⋯H and H⋯O/O⋯H contributions (bottom).

To rationalize our findings, the inter­molecular inter­actions were also analyzed by inspection of Hirshfeld surfaces. The most remarkable feature in the structure packing is that 51.3% of the surface for the individual complex mol­ecule corresponds to contacts involving an H atom and an electronegative one (H⋯Cl, H⋯O and H⋯S with 24.7, 14.8 and 11.8% contributions, respectively). On the other hand, 25.2% correspond to H⋯H contacts, while 8.7% indicate C⋯N (4.4%) and C⋯H (4.3%) contacts. The significance of S⋯O inter­actions is also appreciable. In spite of the relatively small number of such contacts, they deliver a 2.9% contribution to the surface of the mol­ecule. The results of the analysis are summarized in Fig. 7 ▸. These data suggest a potentially higher solubility of **1** in polar solvents compared to copper(II)–famotidine complexes.

## 6. Experimental details

A 30 mL aqueous solution containing 30 mg of pure famotidine (0.089 mmol) at a pH of approximately 3 was mixed with 15 mg of dihydrated copper(II) chloride (0.089 mmol). Upon complete dissolution, the mixture was constantly stirred at 333 K for 45 min before being left to slowly evaporate. Suitable plate-shaped dark-green crystals (yield: 84%) were obtained by slow evaporation of the solvent at room temperature (298 K) for a period of two weeks until the volume reduced to approximately 20 mL.

Elemental analysis for: CuCl_2_C_8_H_14_N_4_O_3_S_2_. Experimental/calculated are: %C 23.71/23.82, %N 13.74/13.58, %H 3.67/3.39 and %S 15.26/15.53.

Elemental analysis for C, N, H, and S was performed on a Thermo Scientific Flash 2000 analyzer. FTIR was recorded as 1% KBr disks on a Shimadzu IR Prestige 21 spectrometer. The thermogram was recorded using a Shimadzu TA-50 equipment, with Pt cells, on a 50 mL min^−1^ air flow in the temperature range of 298–973 K and heating rate of 10 °C min^−1^.

## 7. Refinement

Crystal data, data collection and structure refinement details are summarized in Table 3 ▸. The structure presents a typical example of non-merohedral twinning, the primary signs of which were systematically *F*_o_^2^ >> *F*_c_^2^, but mostly for the reflections with |*h*| = 3*n*. The HKLF5 file was produced after reflection merging. The use of twin law (1 0 0 0 − 1 0 − 0.678 0 − 1), with partial population factors of 0.787 and 0.213, reduced *the R*_1_ [for *I* > 2σ(*I*)] value from 8.34 to 3.23%. All hydrogen atoms were located and refined with isotropic thermal parameters. In the case of NH and carb­oxy­lic OH H atoms, soft restraints in the bond distances were applied [N—H = 0.87 (2) Å; O—H = 0.85 (2) Å].

## Supplementary Material

Crystal structure: contains datablock(s) I. DOI: 10.1107/S2056989026003804/nu2017sup1.cif

Structure factors: contains datablock(s) I. DOI: 10.1107/S2056989026003804/nu2017Isup2.hkl

CCDC reference: 2545262

Additional supporting information:  crystallographic information; 3D view; checkCIF report

## Figures and Tables

**Table 1:** Selected bond lengths (Å) for **1** and related famotidine structures

	FOGVIG08	TAWVIW	This structure
Cu1—N1	–	1.940 (3)	1.959 (2)
Cu1—N3	–	1.926 (3)	1.946 (2)
Cu1—S2	–	2.347 (1)	2.3609 (7)
Cu1—Cl1	–	–	2.2508 (7)
Cu1—Cl2	–	–	2.7164 (8)
N1—C1	1.3234 (4)	1.297 (4)	1.310 (4)
N1—C3	1.3858 (4)	1.399 (4)	1.391 (3)
N2—C1	1.3548 (4)	1.370 (4)	1.373 (4)
N2—C4	1.3390 (3)	1.370 (4)	1.391 (3)
N3—C4	1.3392 (4)	1.286 (5)	1.293 (4)
N4—C4	1.3425 (4)	1.329 (5)	1.346 (4)
C5—C3	1.4919 (4)	1.488 (6)	1.493 (4)
C5—S2	1.8320 (4)	1.831 (4)	1.822 (3)

**Table 2:** Hydrogen-bond geometry (Å, °)

*D*—H⋯*A*	*D*—H	H⋯*A*	*D*⋯*A*	*D*—H⋯*A*
O1—H1*O*⋯O1*W*^i^	0.88 (2)	1.72 (2)	2.577 (3)	165 (4)
O1*W*—H1*W*⋯Cl2	0.85 (5)	2.31 (5)	3.134 (3)	164 (4)
O1*W*—H2*W*⋯Cl1^ii^	0.88 (6)	2.20 (6)	3.075 (3)	175 (5)
N2—H1*N*⋯Cl2^iii^	0.85 (2)	2.32 (2)	3.150 (2)	165 (3)
N3—H2*N*⋯O2^iv^	0.86 (2)	2.20 (2)	3.020 (3)	159 (4)
N4—H4*N*⋯O2^iv^	0.86 (2)	2.27 (3)	3.002 (3)	143 (3)
C5—H5*B*⋯Cl1^v^	0.94 (4)	2.77 (4)	3.560 (3)	142 (3)

Symmetry codes: (i) 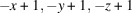; (ii) 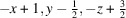; (iii) 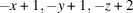; (iv) 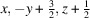; (v) 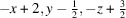.

**Table 3:** Experimental details

Crystal data	
Chemical formula	[CuCl_2_(C_8_H_12_N_4_O_2_S_2_)]·H_2_O
*M*_r_	412.79
Crystal system, space group	Monoclinic, *P*2_1_/*c*
Temperature (K)	100
*a*, *b*, *c* (Å)	6.9498 (2), 12.3041 (2), 17.4871 (3)
β (°)	97.742 (2)
*V* (Å^3^)	1481.71 (6)
*Z*	4
Radiation type	Cu *K*α
μ (mm^−1^)	8.16
Crystal size (mm)	0.1 × 0.08 × 0.04

Data collection	
Diffractometer	XtaLAB Synergy, Dualflex, HyPix
Absorption correction	Multi-scan (*CrysAlis PRO*; Rigaku OD, 2023 ▸)
*T*_min_, *T*_max_	0.480, 0.722
No. of measured, independent and observed [*I* > 2σ(*I*)] reflections	3019, 3019, 2850
*R*_int_	0.048
(sin θ/λ)_max_ (Å^−1^)	0.625

Refinement	
*R*[*F*^2^ > 2σ(*F*^2^)], *wR*(*F*^2^), *S*	0.032, 0.089, 1.06
No. of reflections	3019
No. of parameters	238
No. of restraints	5
H-atom treatment	All H-atom parameters refined
Δρ_max_, Δρ_min_ (e Å^−3^)	0.47, −0.41

Computer programs: *CrysAlis PRO* (Rigaku OD, 2023 ▸), *OLEX2.solve* (Bourhis *et al.*, 2015 ▸), *SHELXL2019/3* (Sheldrick, 2015 ▸) and *OLEX2* (Dolomanov *et al.*, 2009 ▸).

## supplementary crystallographic information

### Dichlorido{3-[(2-guanidinothiazol-4-yl)methylsulfanyl]propanoic acid}copper(II) monohydrate Crystal data

**Table:**

[CuCl_2_(C_8_H_12_N_4_O_2_S_2_)]·H_2_O	*F*(000) = 836
*M**_r_* = 412.79	*D*_x_ = 1.850 Mg m^−^^3^
Monoclinic, *P*2_1_/*c*	Cu *K*α radiation, λ = 1.54184 Å
*a* = 6.9498 (2) Å	Cell parameters from 9793 reflections
*b* = 12.3041 (2) Å	θ = 4.4–79.0°
*c* = 17.4871 (3) Å	µ = 8.16 mm^−^^1^
β = 97.742 (2)°	*T* = 100 K
*V* = 1481.71 (6) Å^3^	Plate, green
*Z* = 4	0.1 × 0.08 × 0.04 mm

### Dichlorido{3-[(2-guanidinothiazol-4-yl)methylsulfanyl]propanoic acid}copper(II) monohydrate Data collection

**Table:**

XtaLAB Synergy, Dualflex, HyPix diffractometer	2850 reflections with *I* > 2σ(*I*)
Detector resolution: 10.0000 pixels mm^-1^	*R*_int_ = 0.048
ω scans	θ_max_ = 74.5°, θ_min_ = 6.3°
Absorption correction: multi-scan (CrysAlisPro; Rigaku OD, 2023)	*h* = −8→8
*T*_min_ = 0.480, *T*_max_ = 0.722	*k* = 0→15
3019 measured reflections	*l* = 0→21
3019 independent reflections	

### Dichlorido{3-[(2-guanidinothiazol-4-yl)methylsulfanyl]propanoic acid}copper(II) monohydrate Refinement

**Table:**

Refinement on *F*^2^	Primary atom site location: structure-invariant direct methods
Least-squares matrix: full	Secondary atom site location: difmapo
*R*[*F*^2^ > 2σ(*F*^2^)] = 0.032	Hydrogen site location: difference Fourier map
*wR*(*F*^2^) = 0.089	All H-atom parameters refined
*S* = 1.06	*w* = 1/[σ^2^(*F*_o_^2^) + (0.0387*P*)^2^ + 2.8736*P*] where *P* = (*F*_o_^2^ + 2*F*_c_^2^)/3
3019 reflections	(Δ/σ)_max_ = 0.001
238 parameters	Δρ_max_ = 0.47 e Å^−^^3^
5 restraints	Δρ_min_ = −0.41 e Å^−^^3^

### Dichlorido{3-[(2-guanidinothiazol-4-yl)methylsulfanyl]propanoic acid}copper(II) monohydrate Special details

**Table:**

Geometry. All esds (except the esd in the dihedral angle between two l.s. planes) are estimated using the full covariance matrix. The cell esds are taken into account individually in the estimation of esds in distances, angles and torsion angles; correlations between esds in cell parameters are only used when they are defined by crystal symmetry. An approximate (isotropic) treatment of cell esds is used for estimating esds involving l.s. planes.
Refinement. Refined as a 2-component twin. 1. Twinned data refinement Scales: 0.7872 (17) 0.2128 (17) 2. Restrained distances O1 H1O 0.85 with sigma of 0.02 3. Restrained distances N2-H1N 0.87 with sigma of 0.02 N3-H2N 0.87 with sigma of 0.02 N4-H3N = N4-H4A 0.87 with sigma of 0.02

### Dichlorido{3-[(2-guanidinothiazol-4-yl)methylsulfanyl]propanoic acid}copper(II) monohydrate Fractional atomic coordinates and isotropic or equivalent isotropic displacement parameters (Å^2^)

**Table:**

	*x*	*y*	*z*	*U*_iso_*/*U*_eq_	
Cu1	0.79627 (6)	0.57610 (3)	0.87235 (2)	0.01287 (12)	
Cl1	0.90647 (11)	0.73350 (5)	0.82907 (4)	0.02036 (17)	
Cl2	0.42378 (10)	0.58562 (5)	0.80174 (4)	0.01679 (15)	
S1	0.71946 (11)	0.26257 (5)	0.99449 (4)	0.01626 (16)	
S2	0.91571 (10)	0.47886 (5)	0.77229 (4)	0.01399 (15)	
O1	0.7376 (4)	0.48044 (18)	0.47891 (13)	0.0327 (6)	
H1O	0.710 (7)	0.520 (3)	0.4370 (18)	0.038 (12)*	
O2	0.7431 (4)	0.64087 (17)	0.53879 (12)	0.0241 (5)	
O1W	0.3475 (4)	0.4351 (2)	0.65624 (14)	0.0268 (5)	
N1	0.7547 (3)	0.42958 (17)	0.91076 (13)	0.0123 (4)	
N2	0.7349 (4)	0.47102 (18)	1.04258 (13)	0.0141 (5)	
N3	0.7696 (4)	0.63279 (18)	0.97427 (14)	0.0141 (5)	
N4	0.7286 (4)	0.63440 (19)	1.10461 (14)	0.0164 (5)	
C1	0.7383 (4)	0.4008 (2)	0.98174 (16)	0.0128 (5)	
C2	0.7330 (4)	0.2443 (2)	0.89740 (17)	0.0166 (6)	
C3	0.7500 (4)	0.3403 (2)	0.86181 (16)	0.0138 (5)	
C4	0.7449 (4)	0.5837 (2)	1.03759 (16)	0.0128 (5)	
C5	0.7572 (5)	0.3621 (2)	0.77828 (16)	0.0163 (5)	
C6	0.8126 (5)	0.5490 (2)	0.68516 (17)	0.0164 (5)	
C7	0.8063 (5)	0.4781 (2)	0.61432 (17)	0.0200 (6)	
C8	0.7594 (4)	0.5430 (2)	0.54087 (17)	0.0166 (6)	
H1W	0.343 (7)	0.476 (4)	0.695 (3)	0.039 (13)*	
H2W	0.280 (8)	0.377 (4)	0.663 (3)	0.050 (14)*	
H1N	0.701 (5)	0.444 (3)	1.0832 (15)	0.021 (9)*	
H2N	0.769 (6)	0.7023 (16)	0.981 (2)	0.026 (10)*	
H3N	0.697 (5)	0.596 (3)	1.1416 (16)	0.016 (9)*	
H4N	0.726 (6)	0.7038 (16)	1.108 (2)	0.020 (9)*	
H2	0.721 (6)	0.178 (4)	0.878 (3)	0.035 (11)*	
H5A	0.639 (6)	0.380 (3)	0.752 (2)	0.025 (10)*	
H5B	0.803 (5)	0.301 (3)	0.754 (2)	0.017 (9)*	
H6A	0.687 (5)	0.573 (3)	0.690 (2)	0.010 (8)*	
H6B	0.903 (6)	0.604 (3)	0.683 (2)	0.029 (10)*	
H7A	0.713 (6)	0.418 (3)	0.614 (2)	0.030 (11)*	
H7B	0.921 (7)	0.443 (4)	0.613 (3)	0.042 (13)*	

### Dichlorido{3-[(2-guanidinothiazol-4-yl)methylsulfanyl]propanoic acid}copper(II) monohydrate Atomic displacement parameters (Å^2^)

**Table:**

	*U*^11^	*U*^22^	*U*^33^	*U*^12^	*U*^13^	*U*^23^
Cu1	0.0190 (2)	0.0086 (2)	0.0116 (2)	−0.00177 (15)	0.00407 (16)	0.00069 (13)
Cl1	0.0301 (4)	0.0117 (3)	0.0212 (3)	−0.0060 (3)	0.0104 (3)	0.0006 (2)
Cl2	0.0181 (3)	0.0175 (3)	0.0152 (3)	0.0013 (2)	0.0040 (2)	0.0031 (2)
S1	0.0271 (4)	0.0088 (3)	0.0131 (3)	−0.0013 (2)	0.0033 (3)	0.0026 (2)
S2	0.0182 (3)	0.0125 (3)	0.0117 (3)	0.0004 (2)	0.0032 (2)	0.0013 (2)
O1	0.0666 (18)	0.0163 (11)	0.0150 (11)	0.0059 (11)	0.0041 (11)	0.0004 (8)
O2	0.0421 (13)	0.0119 (10)	0.0172 (10)	0.0018 (9)	−0.0001 (9)	0.0015 (8)
O1W	0.0348 (13)	0.0255 (12)	0.0187 (11)	−0.0063 (10)	−0.0012 (9)	0.0006 (10)
N1	0.0149 (11)	0.0096 (10)	0.0124 (11)	0.0014 (8)	0.0019 (8)	−0.0001 (8)
N2	0.0214 (12)	0.0102 (11)	0.0109 (11)	−0.0007 (9)	0.0034 (9)	0.0014 (8)
N3	0.0205 (12)	0.0082 (10)	0.0139 (11)	−0.0012 (9)	0.0029 (9)	−0.0016 (8)
N4	0.0249 (13)	0.0120 (11)	0.0125 (11)	0.0003 (9)	0.0038 (9)	−0.0001 (9)
C1	0.0147 (12)	0.0103 (12)	0.0131 (13)	0.0001 (10)	0.0010 (10)	0.0015 (10)
C2	0.0259 (15)	0.0104 (13)	0.0140 (13)	0.0002 (11)	0.0042 (11)	−0.0006 (10)
C3	0.0150 (13)	0.0103 (12)	0.0160 (13)	0.0004 (10)	0.0021 (10)	−0.0006 (10)
C4	0.0128 (12)	0.0101 (12)	0.0149 (13)	0.0000 (10)	0.0000 (10)	−0.0001 (10)
C5	0.0224 (14)	0.0099 (12)	0.0169 (14)	0.0006 (11)	0.0038 (11)	−0.0016 (10)
C6	0.0222 (14)	0.0125 (13)	0.0147 (13)	0.0026 (11)	0.0032 (11)	0.0020 (10)
C7	0.0333 (17)	0.0129 (13)	0.0143 (14)	0.0034 (12)	0.0051 (12)	0.0018 (11)
C8	0.0214 (14)	0.0135 (13)	0.0156 (14)	0.0013 (11)	0.0047 (11)	0.0002 (10)

### Dichlorido{3-[(2-guanidinothiazol-4-yl)methylsulfanyl]propanoic acid}copper(II) monohydrate Geometric parameters (Å, º)

**Table:**

Cu1—N3	1.946 (2)	N2—H1N	0.847 (19)
Cu1—N1	1.959 (2)	N3—C4	1.293 (4)
Cu1—Cl1	2.2508 (7)	N3—H2N	0.863 (19)
Cu1—S2	2.3609 (7)	N4—C4	1.346 (4)
Cu1—Cl2	2.7164 (8)	N4—H3N	0.852 (19)
S1—C1	1.722 (3)	N4—H4N	0.856 (19)
S1—C2	1.728 (3)	C2—C3	1.348 (4)
S2—C6	1.812 (3)	C2—H2	0.89 (5)
S2—C5	1.822 (3)	C3—C5	1.493 (4)
O1—C8	1.321 (4)	C5—H5A	0.91 (4)
O1—H1O	0.881 (19)	C5—H5B	0.94 (4)
O2—C8	1.210 (4)	C6—C7	1.511 (4)
O1W—H1W	0.85 (5)	C6—H6A	0.94 (4)
O1W—H2W	0.88 (6)	C6—H6B	0.92 (4)
N1—C1	1.310 (4)	C7—C8	1.510 (4)
N1—C3	1.391 (3)	C7—H7A	0.98 (4)
N2—C1	1.373 (4)	C7—H7B	0.91 (5)
N2—C4	1.391 (3)		
			
N3—Cu1—N1	88.81 (10)	C3—C2—S1	111.1 (2)
N3—Cu1—Cl1	94.49 (7)	C3—C2—H2	130 (3)
N1—Cu1—Cl1	168.47 (7)	S1—C2—H2	119 (3)
N3—Cu1—S2	161.24 (8)	C2—C3—N1	113.9 (2)
N1—Cu1—S2	82.57 (7)	C2—C3—C5	128.7 (3)
Cl1—Cu1—S2	90.99 (3)	N1—C3—C5	117.3 (2)
N3—Cu1—Cl2	101.45 (8)	N3—C4—N4	124.4 (2)
N1—Cu1—Cl2	91.14 (7)	N3—C4—N2	122.2 (3)
Cl1—Cu1—Cl2	99.01 (3)	N4—C4—N2	113.4 (2)
S2—Cu1—Cl2	95.37 (3)	C3—C5—S2	107.35 (19)
C1—S1—C2	89.21 (13)	C3—C5—H5A	113 (3)
C6—S2—C5	104.49 (14)	S2—C5—H5A	107 (3)
C6—S2—Cu1	104.08 (10)	C3—C5—H5B	111 (2)
C5—S2—Cu1	94.67 (9)	S2—C5—H5B	111 (2)
C8—O1—H1O	110 (3)	H5A—C5—H5B	108 (3)
H1W—O1W—H2W	108 (5)	C7—C6—S2	112.3 (2)
C1—N1—C3	111.8 (2)	C7—C6—H6A	109 (2)
C1—N1—Cu1	127.55 (19)	S2—C6—H6A	110 (2)
C3—N1—Cu1	120.56 (18)	C7—C6—H6B	109 (3)
C1—N2—C4	124.9 (2)	S2—C6—H6B	101 (3)
C1—N2—H1N	116 (3)	H6A—C6—H6B	114 (3)
C4—N2—H1N	118 (3)	C8—C7—C6	111.8 (2)
C4—N3—Cu1	131.06 (19)	C8—C7—H7A	109 (2)
C4—N3—H2N	110 (3)	C6—C7—H7A	112 (2)
Cu1—N3—H2N	118 (3)	C8—C7—H7B	109 (3)
C4—N4—H3N	118 (2)	C6—C7—H7B	112 (3)
C4—N4—H4N	122 (3)	H7A—C7—H7B	103 (4)
H3N—N4—H4N	119 (4)	O2—C8—O1	123.7 (3)
N1—C1—N2	125.2 (2)	O2—C8—C7	124.1 (3)
N1—C1—S1	114.0 (2)	O1—C8—C7	112.2 (2)
N2—C1—S1	120.8 (2)		
			
C3—N1—C1—N2	−178.9 (3)	Cu1—N1—C3—C5	−6.2 (3)
Cu1—N1—C1—N2	4.5 (4)	Cu1—N3—C4—N4	−180.0 (2)
C3—N1—C1—S1	0.9 (3)	Cu1—N3—C4—N2	0.8 (4)
Cu1—N1—C1—S1	−175.70 (14)	C1—N2—C4—N3	−4.0 (4)
C4—N2—C1—N1	1.2 (4)	C1—N2—C4—N4	176.7 (3)
C4—N2—C1—S1	−178.6 (2)	C2—C3—C5—S2	−146.7 (3)
C2—S1—C1—N1	−0.5 (2)	N1—C3—C5—S2	35.7 (3)
C2—S1—C1—N2	179.3 (2)	C6—S2—C5—C3	−146.7 (2)
C1—S1—C2—C3	−0.1 (2)	Cu1—S2—C5—C3	−40.81 (19)
S1—C2—C3—N1	0.6 (3)	C5—S2—C6—C7	−59.6 (3)
S1—C2—C3—C5	−177.0 (2)	Cu1—S2—C6—C7	−158.3 (2)
C1—N1—C3—C2	−1.0 (4)	S2—C6—C7—C8	−168.4 (2)
Cu1—N1—C3—C2	175.9 (2)	C6—C7—C8—O2	5.1 (5)
C1—N1—C3—C5	177.0 (2)	C6—C7—C8—O1	−174.7 (3)

### Dichlorido{3-[(2-guanidinothiazol-4-yl)methylsulfanyl]propanoic acid}copper(II) monohydrate Hydrogen-bond geometry (Å, º)

**Table:**

*D*—H···*A*	*D*—H	H···*A*	*D*···*A*	*D*—H···*A*
O1—H1*O*···O1*W*^i^	0.88 (2)	1.72 (2)	2.577 (3)	165 (4)
O1*W*—H1*W*···Cl2	0.85 (5)	2.31 (5)	3.134 (3)	164 (4)
O1*W*—H2*W*···Cl1^ii^	0.88 (6)	2.20 (6)	3.075 (3)	175 (5)
N2—H1*N*···Cl2^iii^	0.85 (2)	2.32 (2)	3.150 (2)	165 (3)
N3—H2*N*···O2^iv^	0.86 (2)	2.20 (2)	3.020 (3)	159 (4)
N4—H4*N*···O2^iv^	0.86 (2)	2.27 (3)	3.002 (3)	143 (3)
C5—H5*B*···Cl1^v^	0.94 (4)	2.77 (4)	3.560 (3)	142 (3)

Symmetry codes: (i) −*x*+1, −*y*+1, −*z*+1; (ii) −*x*+1, *y*−1/2, −*z*+3/2; (iii) −*x*+1, −*y*+1, −*z*+2; (iv) *x*, −*y*+3/2, *z*+1/2; (v) −*x*+2, *y*−1/2, −*z*+3/2.
